# The clinical efficacy of intravenous IgM-enriched immunoglobulin (pentaglobin) in sepsis or septic shock: a meta-analysis with trial sequential analysis

**DOI:** 10.1186/s13613-019-0501-3

**Published:** 2019-02-06

**Authors:** Jie Cui, Xuxia Wei, Haijin Lv, Yuntao Li, Ping Li, Zhen Chen, Genglong Liu

**Affiliations:** 1 Head and Neck Surgery, Affiliated Cancer Hospital & Institute of Guangzhou Medical University, Guangzhou, 510095 Guangdong Province People’s Republic of China; 2 Department of Pathology, Affiliated Cancer Hospital & Institute of Guangzhou Medical University, Guangzhou, 510095 Guangdong Province People’s Republic of China; 3 Surgical Intensive Care Unit, The Third Affiliated Hospital of Sun Yat-sen University, Guangzhou, 510630 Guangdong Province People’s Republic of China; 4 Intensive Care Unit, Shunde Hospital, Southern Medical University, Foshan, 528300 Guangdong Province People’s Republic of China; 5 Nursing Department, Shaodong County People’s Hospital, Shaodong, 422800 Hunan Province People’s Republic of China; 6 Department of Anesthesia, GuangDong Provincial Hospital of Chinese Medicine, Guangzhou, 510120 Guangdong Province People’s Republic of China

**Keywords:** Sepsis, IgM-enriched immunoglobulin, Mortality, Trial sequential analysis

## Abstract

**Background:**

Sepsis is characterized by a complex immune response. This meta-analysis evaluated the clinical effectiveness of intravenous IgM-enriched immunoglobulin (IVIgGM) in patients with sepsis and septic shock.

**Methods:**

Four databases, PubMed, the Cochrane Library, the ISI Web of Knowledge, and Embase, were systematically searched from inception to June 2018 to update the 2013 edition of the Cochrane review by two investigators, who independently selected studies, extracted relevant data, and evaluated study quality. Data were subjected to a meta-analysis and trial sequential analysis (TSA) for the primary and secondary outcomes. Level of evidence was evaluated using the Grading of Recommendations Assessment, Development, and Evaluation (GRADE) scale.

**Results:**

Nineteen studies comprising 1530 patients were included in this meta-analysis. Pooled analyses showed that the use of IVIgGM reduced the mortality risk of septic patients (relative risk 0.60; 95% confidence interval [CI] 0.52–0.69, *I*^2^ = 0%). TSA showed that IVIgGM had a significant effect on mortality. Additionally, the meta-analysis suggested that use of IVIgGM shortened length of mechanical ventilation (mean difference − 3.16 days; 95% CI − 5.71 to − 0.61 days) and did not shorten length of stay in the intensive care unit (mean difference − 0.38 days; 95% CI − 3.55 to 2.80 days). The GRADE scale showed that the certainty of the body of evidence was low for both benefits and IVIgGM.

**Conclusion:**

Administration of IVIgGM to adult septic patients may be associated with reduced mortality. Treatment effects tended to be smaller or less consistent when including only those studies deemed adequate for each indicator. The available evidence is not clearly sufficient to support the widespread use of IVIgGM in the treatment of sepsis.

*Trial registration* PROSPERO registration number: CRD42018084120. Registered on 11 February 2018.

**Electronic supplementary material:**

The online version of this article (10.1186/s13613-019-0501-3) contains supplementary material, which is available to authorized users.

## Introduction

Sepsis and the related syndrome of multiple organ failure remain worldwide problems, with high mortality and morbidity rates [1]. The standard surviving sepsis campaign (SSC) approach, including early eradication of septic foci, administration of anti-infective agents, and maintenance of hemodynamic stability through fluid administration and vasopressors, remains the cornerstone of treatment for sepsis and, in particular, septic shock [2]. However, sepsis is a complex syndrome in that different types of microorganisms (e.g., bacteria, fungi) that vary in virulence (e.g., endotoxin production) and resistance to antibiotics may infect one or more body sites in patients with varying comorbidities. These patients, in turn, may vary widely in their responses to infection (e.g., hyper-inflammation, immune paralysis) and treatments.

The recent Third International Consensus (Sepsis-3) defined sepsis as a life-threatening organ dysfunction caused by a dysregulated host response to infection [3]. The host response to an infection consists of an exert inflammatory storm and concurrent immunosuppression, characterized by overwhelming promotive tissue damage, down-regulation of activating cell-surface molecules, T cell exhaustion and increased apoptosis of immune cells [4]. These immunodisturbances cause a profound dysfunction in innate/adaptive immune responses [5] and seem to play a vital role in patient outcomes, particularly in older patients and those with preexisting immune dysfunction. Since only anti-inflammation therapies failed to save sepsis lives, an increased use of strategies designed to balance the immune system seems more reasonable.

Polyclonal intravenous immunoglobulins (IVIG), which have pleiotropic effects on inflammatory and immune mechanisms, have been proposed as adjuvant therapy to modulate both pro- and anti-inflammatory processes [6]. In 2016, SSC guidelines suggested against IVIG use in sepsis, which was based on weak evidence of efficacy from previous studies [7]. However, results from recent trials and systematic meta-analyses indicate that intravenous IgM-enriched immunoglobulins (IVIgGM) may be effective in septic patients [8–10]. The present study therefore conducted a meta-analysis with trial sequential analysis (TSA) to evaluate the clinical effectiveness of IVIgGM in septic patients, with a view to helping guide clinicians in making treatment decisions.

## Methods

This meta-analysis was performed according to the Cochrane Handbook for Systematic Reviews of Interventions and presented based on Preferred Reporting Items for Systematic Reviews and Meta-analyses guidelines (PRISMA) [11]. The review protocol was registered at the PROSPERO registry of systematic reviews in February 2018 (Registry Number: CRD42018084120).

### Data sources

A systematic search of the PubMed, Cochrane Library, ISI Web of Knowledge, and Embase databases was conducted to update the 2013 edition of the Cochrane review [10] from inception to June 2018. Since its publication, 5 trials, including 3 randomized controlled trials (RCTs) [12–14] and 2 retrospective cohorts [8, 9], regarding the use of IVIgGM in sepsis have been published. The search strategy consisted of: (iviggma [All Fields] OR (igm [All Fields] AND enriched [All Fields]) OR (pentaglobulin [Supplementary Concept] OR pentaglobulin [All Fields] OR pentaglobin [All Fields])) AND (sepsis [MeSH Terms] OR sepsis [All Fields]). There were no language restrictions. Additional studies were identified by reviewing the reference lists of relevant articles.

### Eligibility criteria

Two reviewers independently evaluated studies to determine their eligibility for inclusion in the meta-analysis. In cases of disagreement, a consensus was reached by discussion or by consultation with a third reviewer. Trials were included if they: (1) compared IVIgGM with a placebo or another treatment group; (2) enrolled adult patients aged ≥ 18 years with sepsis; and (3) provided mortality data. Publications were excluded if they described irrelevant research or animal experiments. Also excluded were review articles, meeting abstracts, studies of pediatric patients, and studies with insufficient information (e.g., absence of mortality data), even after contacting the corresponding authors.

### Data extraction

Using standard forms, two reviewers independently extracted the data from each eligible study, including lead author, year of publication, study design, number of participating centers, sepsis severity, number of patients, mean ages of patients in the treatment and control groups, duration of treatment, daily dose of medication, type of control, baseline severity scores (e.g., Acute Physiology and Chronic Health Evaluation [APACHE] II Score; Sequential Organ Failure Assessment [SOFA]; and Sepsis Score) for the treated and control groups. Also follow-up period, number of deaths due to sepsis and the mean ± standard deviation or median length of mechanical ventilation and length of stay (LOS) in the intensive care unit (ICU) were recorded. If a meta-analysis mentioned that unpublished data were provided by the primary authors, these data were extracted from the forest plots of the meta-analysis and the original articles were reviewed to confirm whether those trials met the inclusion criteria of this meta-analysis. If these data were among the outcomes of interest, they were pooled with data from primary trials. Whenever possible, outcome data were separately extracted for each subgroup. The duration of treatment, daily dose, total dose, APACHE II or SOFA Score and publication year were recoded as “low” or “high” based on whether they fell below or above the median value of the entire set of studies. The primary outcome was all-cause mortality, including in-ICU mortality, 12-day mortality, 28-day mortality, 30-day mortality, 42-day mortality, and 70-day mortality. The secondary outcome was length of mechanical ventilation and LOS in an ICU.

### Quality assessment

The methodological quality of randomized controlled trials (RCTs) was assessed by the Cochrane risk of bias tool [15]. Each quality item was graded as low risk, high risk, or unclear risk. The seven items used to evaluate bias in each trial included the randomization sequence generation, allocation concealment, blinding of participants and personnel, blinding of outcome assessments, incomplete outcome data, selective reporting, and other biases. The overall risk of bias for each study was evaluated and rated as “low” when the risk of bias was low in all key domains; “unclear” when the risk of bias was low or unclear in all key domains; and “high” when the risk of bias was high in one or more key domains. The methodological quality of observational studies was assessed by the Newcastle–Ottawa scale, which consists of eight items evaluating the quality of observational studies, such as their selection, comparability, and outcome. Each study was given a Newcastle–Ottawa scale score of 0–9 (allocated as stars), with observational studies receiving ≥ 6 stars considered to be of high quality.

### Statistical analyses

Dichotomous data were expressed as risk ratio (RR) and continuous outcomes as weighted mean difference (WMD), both with their 95% confidence intervals (CI). Continuous variables were reported as mean (± SD) or median (interquartile range). To convert a median (interquartile range) to a mean (standard deviation), we used the formulas accepted in the literature [16]. Chi-squared tests and the *I*^2^ statistic were used to measure statistical heterogeneity. A *P* value < 0.05 or *I*^2^ > 50% was considered indicative of substantial heterogeneity, leading to the application of a random effects model to estimate the summary RR, WMD and 95% CI; otherwise, a fixed effects model was applied.

To evaluate whether the association between administration of IVIgGM and mortality was modified by clinical characteristics, subgroups were specified based on treatment duration (> 3 vs. ≤ 3 days), daily dose (> 0.25 vs. ≤ 0.25 g/kg), total dose (≥ 0.9 vs. < 0.9 g/kg), type of control intervention (placebo vs. human albumin solution), sepsis severity (sepsis vs. severe sepsis or septic shock), severity score(low APACHE II or SOFA Score vs. high APACHE II or SOFA Score), follow-up duration in the ICU (> 28 vs. ≤ 28 days),study design (RCT vs. cohort study) and year of publication (before 2005 vs. 2005 onward). Analysis was performed to assess whether the difference between the subgroups was statistically significant.

Meta-regression analysis (MRA) was performed to explore the potential effects of heterogeneity and confounders on outcomes. Factors considered variables included year of publication, number of participating centers, number of patients, mean age, duration of treatment, daily dose, total dose, mortality rates of the IVIgGM and mortality rates of control groups. Publication bias was assessed by examining funnel plots when ≥ 10 trials reported the primary outcomes.

All meta-analyses were performed using RevMan version 5.3 (Cochrane Collaboration) and Stata version 14.0 (Stata Corporation, College Station, Tex). All tests were 2-tailed, and *P* < 0.05 was considered statistically significant.

### Grading the quality of evidence

Two investigators independently assessed the quality of evidence for outcomes using the Grading of Recommendations Assessment, Development, and Evaluation (GRADE). Each outcome was classified as having evidence of high, moderate, low, or very low quality based on risk of bias, inconsistency, indirectness, imprecision, and publication bias. GRADE Pro-version 3.6 software was used for these analyses.

### Trial sequential analysis

TSA was used to evaluate the cumulative effect of randomized trials on mortality. In this procedure, *Z*-curves were constructed for the primary outcome, and an alpha value at a conventional threshold was used to determine significance. Adjusted significance trial sequential monitoring boundaries were constructed using the O’Brien-Fleming alpha spending method, with the assumption that significance testing may have been performed each time a new trial was sequentially added to the meta-analysis. For the TSA, the required information size was calculated based on a relative risk reduction of 20% in outcomes. The type I error (*α*) was set at 0.05 or 0.01, and the power (1 − *β*) at 0.80. The control event rates were calculated from the control group. TSA was performed using TSA version 0.9 beta software (http://www.ctu.dk/tsa) [17].

## Results

### Identification of studies

**Fig. 1:**
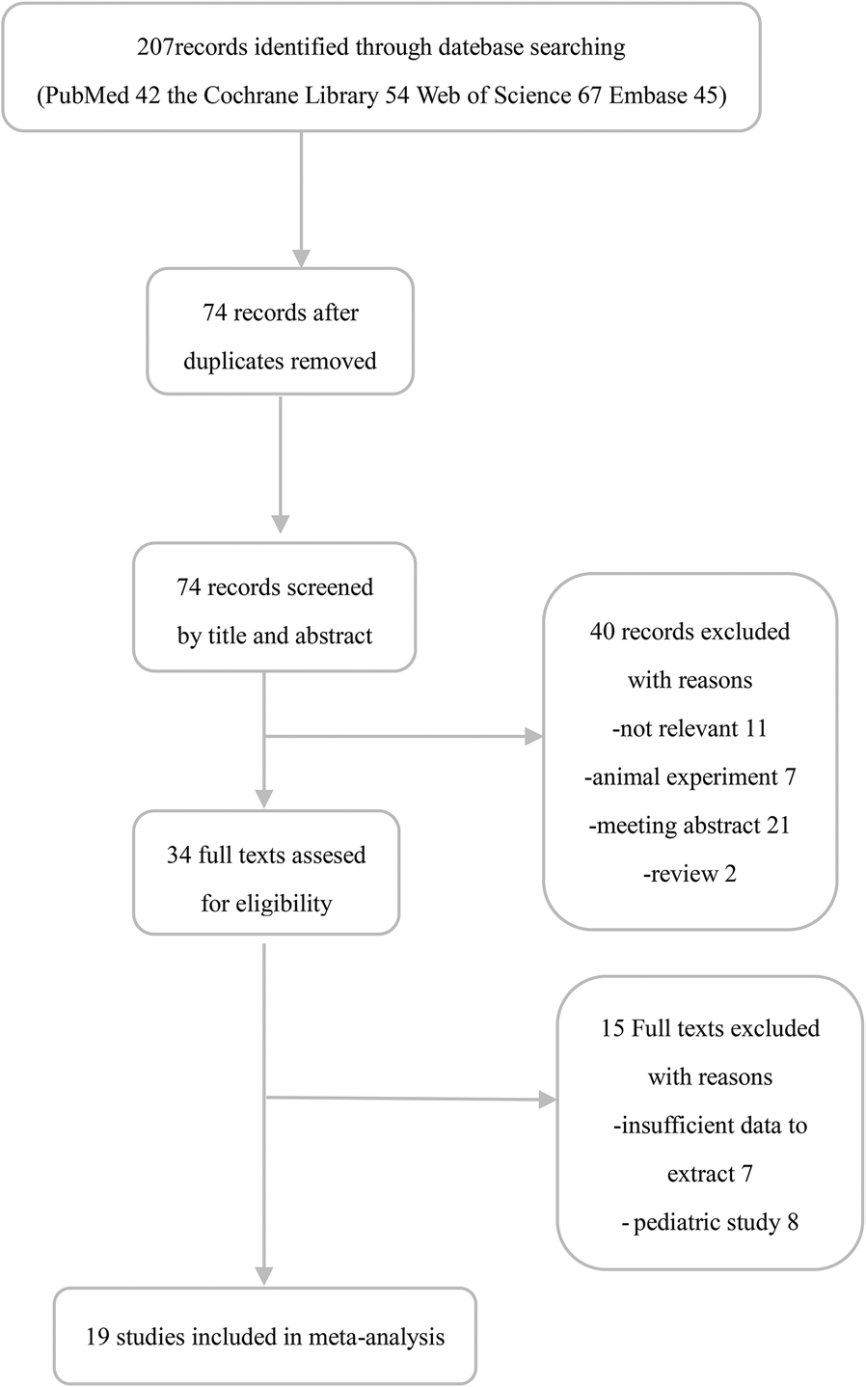
Flow diagram of literature search and selection process of the studies

The flow chart of the study selection procedure is shown in Fig. 1. The initial search identified 41 studies in PubMed, 54 in the Cochrane Library, 67 in the ISI Web of Knowledge, and 45 in Embase. After removing 133 duplicates, the titles and abstracts of the remaining 74 studies were screened. Forty studies were eliminated after reading their titles and abstracts, and 34 articles were scrutinized by reading their full texts. Seven articles were excluded because of insufficient data and seven were excluded because their study population consisted of children. Ultimately, 19 studies [8, 9, 12–14, 18–31] fulfilled our eligibility criteria and were included in the final meta-analyses.

### Characteristics of the included studies

Characteristics of the included studies are shown in Table 1. The 18 studies included 15 RCTs [12–14, 16, 20–30] and four observational studies [8, 9, 19, 31], with a total of 1530 patients, and were published from 1986 to 2018. Fourteen studies (21%) were single-center and five (79%) were multi-center. The number of patients per study varied from 29 to 206. Mean age of patients in 15 of the included studies varied between 42 and 71.7 years, whereas mean age was not reported for four studies [21, 23, 24, 29]. Dosing regimens varied widely, with duration of treatment ranging from 1.5 to 5 days; one study did not report treatment duration [27], whereas 13 (68.4%) reported that patients were treated for 3 days. Average daily dose ranged from 0.15 to 0.35 g/kg/day, although two studies did not report this parameter [14, 19, 27]. Human albumin solution (HAS) was used as a control intervention in five studies, whereas no treatment was provided for the control arm in 14 studies. Follow-up periods varied, including follow-up in the ICU and after 12, 28, 30, 42 and 70 days. Additional file 1: Table S1 details the primary and secondary outcomes of the studies included in the meta-analysis.

**Table 1:** Characteristics of the studies included in the meta-analysis

References	Study design	Participating centers (*N*.)	Patients (*N*.)	Mean age IVIgGM/control (years)	Duration of treatment (days)	Daily dose (g/kg)	Control	Severity score (IgM/control)	Follow-up (days)
Behre et al. [18]	RCT	2	52	50/55	3	0.31	5% HAS	NR/NR	28
Brunne et al. [12]	RCT	1	38	61/66	3	0.25	HAS	SOFA score 11(4)/11(5)	28
Buda et al. [19]	ROS	1	66	62.9/68.6	3	0.25	Placebo	APACHE II 20.5(5.8)/21.5(5.4)	70
Cavazzuti et al. [8]	ROS	1	168	68.9/71.7	3	0.25	Placebo	SOFA score 9.5(3.3)/8.6(3.6)	30
Giamarellos-Bourboulis et al. [9]	POS	63	200	51.9/54.2	5	> 0.25	Placebo	APACHE II 19.6(6.9)/20.7(6.6)	28
Hentrich et al. [20]	RCT	6	206	48.8/51.0	3	0.31	HAS	NR/NR	28
Just et al. [21]	RCT	1	29	NR/NR	1.5	NR	Placebo	NR/NR	ICU
Karatzas et al. [22]	RCT	1	68	50.5/50.7	3	0.25	Placebo	APACHE II 21.3 (7.2)/23.5 (7.9)	28
Reith et al. [23]	RCT	1	67	NR/NR	3	0.2	Placebo	NR/NR	ICU
Rodriguez et al. [24]	RCT	1	37	NR/NR	5	0.35	5% HAS	NR/NR	30
Rodriguez et al. [25]	RCT	7	56	61.3/65.9	5	0.35	5% HAS	APACHE II 16.1 (5.9)/15.2 (6.1)	ICU
Schedel et al. [26]	RCT	1	55	46/37	3	0.285	Placebo	APACHE II 30/24	42
Spannbrucker et al. [27]	RCT	1	50	50.8/54.5	3	0.15	Placebo	NR/NR	12
Toth et al. [13]	RCT	1	33	56/60	3	0.25	Placebo	APACHE II 26 (5.25)/25 (5.5)	28
Tugrul et al. [28]	RCT	1	42	42/49.3	3	0.25	Placebo	APACHE II 10.5 (4.6)/14 (8.5)	28
Vogel et al. [29]	RCT	1	50	NR/NR	NR	NR	Placebo	NR/NR	ICU
Welte et al. [14]	RCT	3	160	63.7/65.5	5	NR	Placebo	SOFA score 9.7(3.8)/10.8(3.5)	28
Wesoly et al. [30]	RCT	1	35	44.7/54.8	3	0.25	Placebo	Sepsis score 14.8 (2.5)/16.3 (3.6)	ICU
Yavuz et al. [31]	ROS	1	118	54.5/59.5	3	0.25	Placebo	APACHE II 27.1/27	28

*N* number, *NR* not reported, *HAS* human albumin solution, *RCT* randomized controlled trial, *ICU* intensive care medicine, *ROS* retrospective observational study, *POS* prospective observational study

### Methodological quality of included studies

Additional file 2: Table S2 shows the quality assessment of the RCTs. Four had a high risk of bias because of undefined random methods. Five studies had a moderate risk of bias because participants and personnel were not blinded. The remaining study had a low risk of bias. Additional file 3: Table S3 shows the quality assessment of the four observational studies. Three studies each had scores of nine points, and the fourth had a score of eight points.

### Primary outcomes

**Fig. 2:**
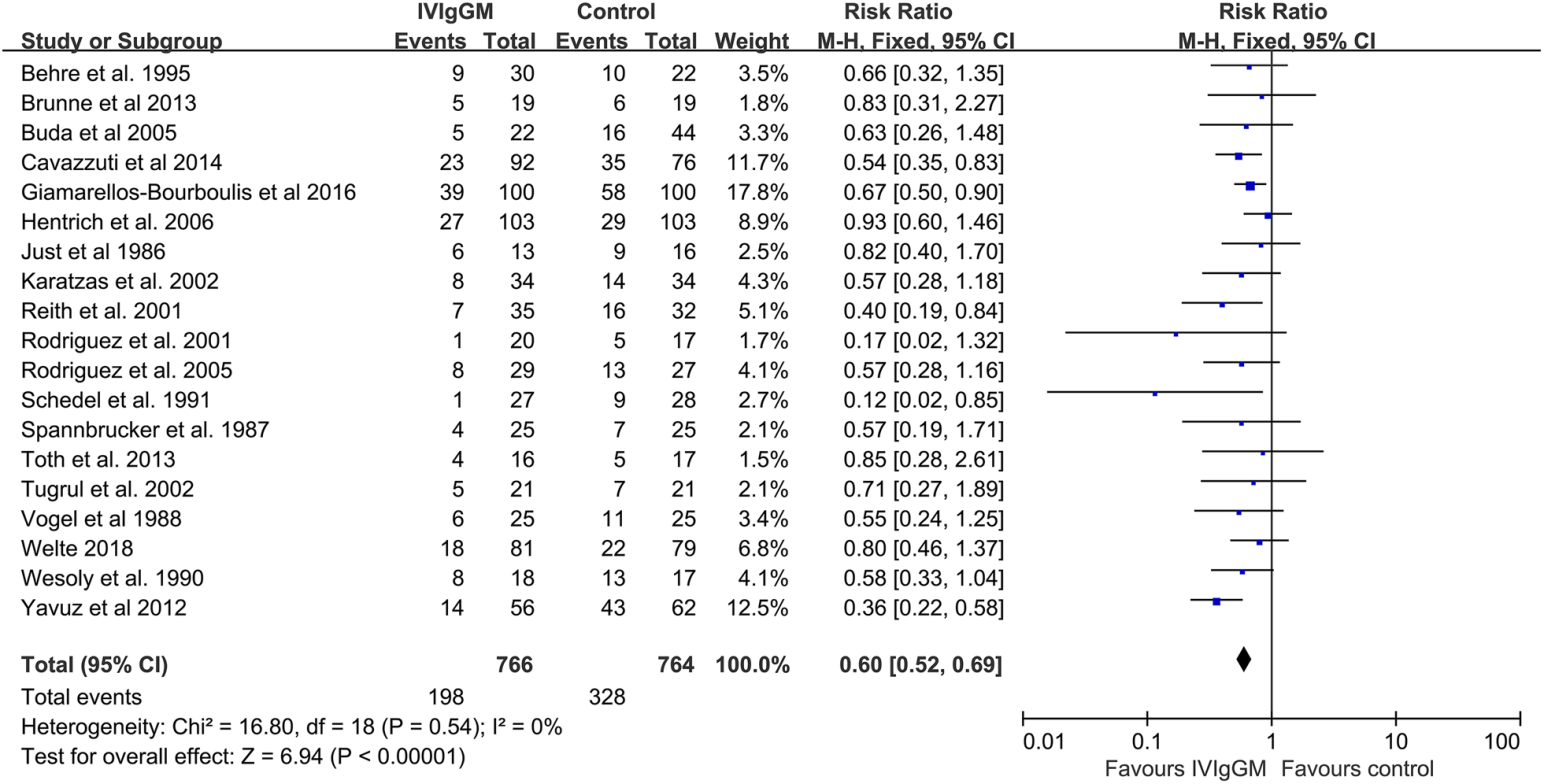
Forest plot showing the overall effect of IVIgGM on mortality in adults with sepsis

**Fig. 3:**
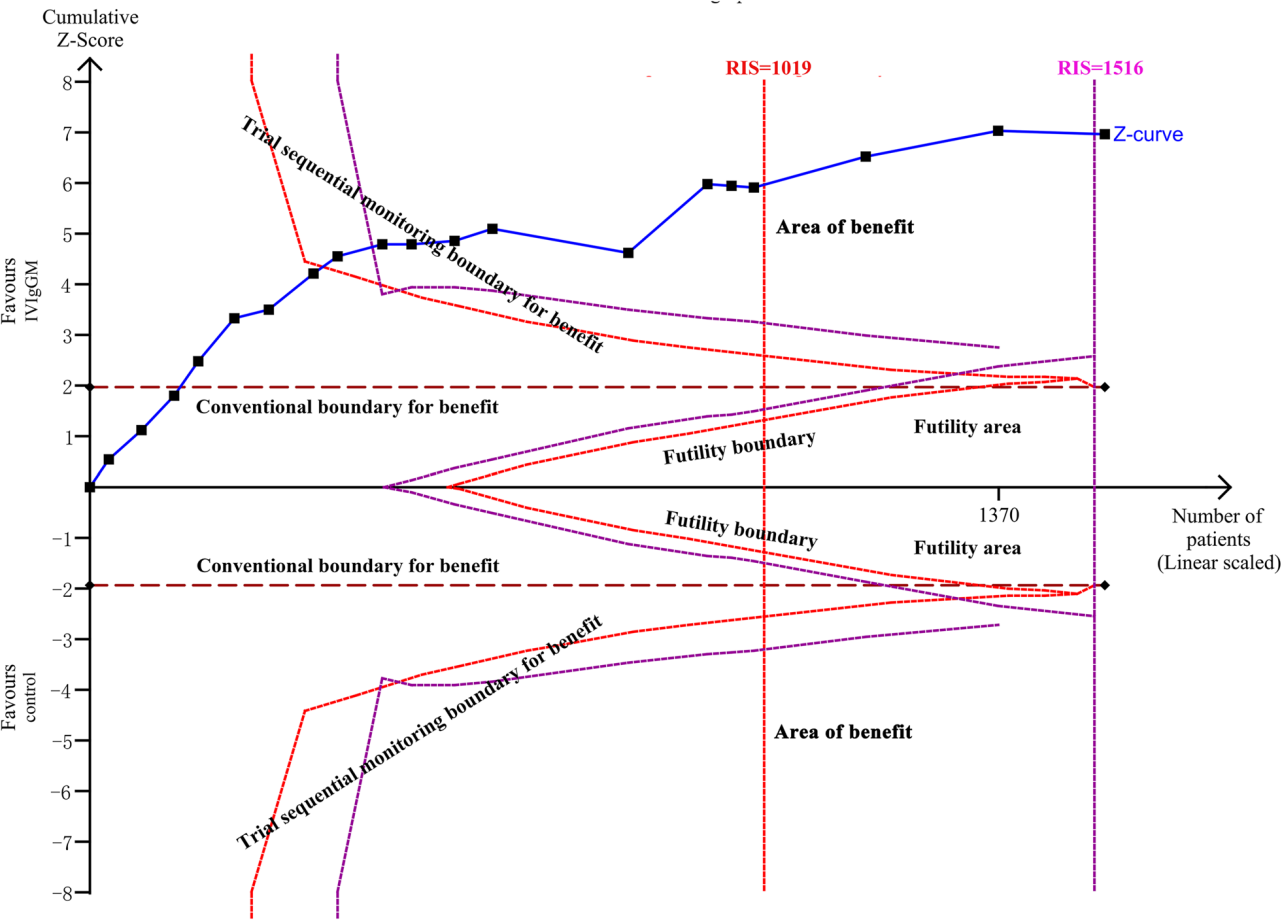
Trial sequential analysis for mortality in trials: a relative risk of 0.60, two-sided boundary, incidence of 25% in IVIgGM group, incidence of 42.7% in control group, a low bias estimated relative risk reduction of 80%, α of 5%, power of 80% were set. The required information size was calculated as 1019. Z-curve has across-trial sequential monitoring boundary for benefit

Pooled estimates indicated that mortality rates were significantly lower in patients who received IVIgGM than in their respective control groups (relative risk [RR] 0.60; 95% confidence interval [CI] 0.52–0.69) (Fig. 2). Statistical homogeneity was met (*I*^2^ = 0%), and a fixed effects model was used to estimate the summary RR, and 95% CI. TSA results showed that the cumulative *Z*-curve crossed the conventional boundary and the trial sequential monitoring boundary (Fig. 3). These results indicate that this evidence is sufficient and conclusive, and that further trials are not required.

**Fig. 4:**
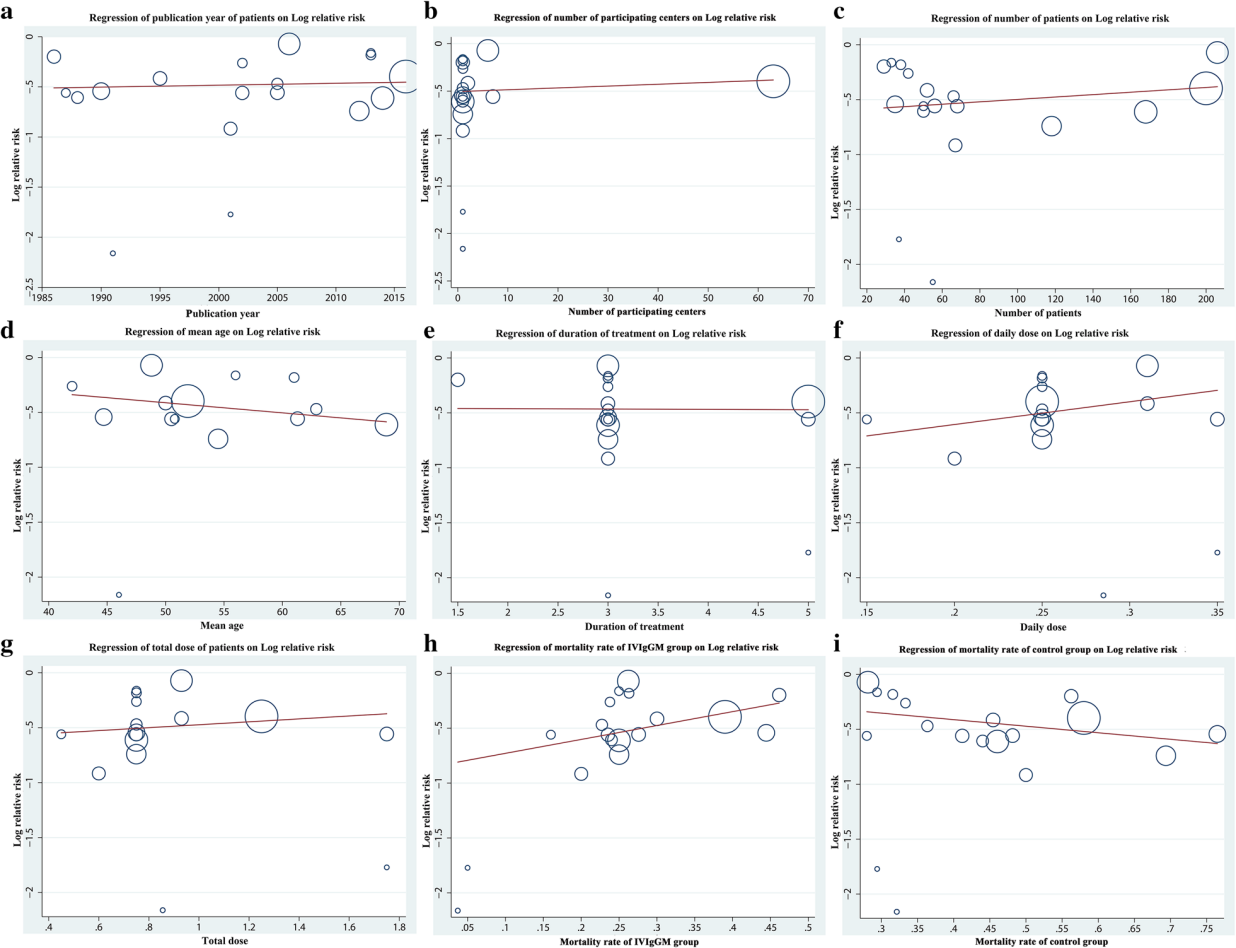
Random-effects meta-regression analyses showing the relationship between the study effect size and **a** publication year, **b** number of participating centers, and mortality rates of the IVIgGM and control groups. **c** Number of patients, **d** mean age, **e** duration of treatment, **f** daily dose, **h** total dose, **i** mortality rates of the IVIgGM, **j** mortality rates of the control groups. The size of the circles is inversely proportional to the size of the result study variance, so that more precise studies have larger circles

Separate meta-analyses performed for sepsis and severe sepsis or septic shock subgroups showed similar significant effects (sepsis: RR = 0.60[0.46, 0.80], *I*^2^ = 0; severe sepsis or septic shock: RR = 0.60 [0.51, 0.71], *I*^2^ = 36) (Additional file 4: Figure S1). Likewise, Subgroup analyses based on severity score. In low APACHE II or SOFA subgroup, the pooled RR was 0.65 (95% CI 0.53–0.80; *I*^2^ = 0). In high APACHE II or SOFA subgroup, the pooled RR was 0.46(95% CI 0.34–0.63; *I*^2^ = 20) (Additional file 5: Figure S2). Additionally, subgroup analyses based on type of control intervention were performed. Compared with placebo, the pooled RR for IVIgGM was 0.57 (95% CI 0.48–0.67; *I*^2^ = 0). However, compared with HAS, the pooled RR was 0.74 (95% CI 0.54–1.01; *I*^2^ = 0). Other analyses showed that the results were generally consistent, regardless of duration of treatment, daily dose, total dose, follow-up duration, study design and year of publication (Table 2). A meta-regression analysis indicated that no variables significantly altered effect size (Fig. 4), whereas a trend toward a greater and more consistent decrease in mortality was seen among studies involving older patients.

**Table 2:** Results of subgroup analysis based on different standards

	*K*	*N*	RR [95% CI]	*P*	Study heterogeneity				*P* (between-group comparison)
					Chi^2^	*df*	*I*^2^ (%)	*P*	
Duration of treatment									0.40
≤ 3 days	14	1027	0.58 [0.48, 0.69]	< 0.001	13.75	13	5	0.39	
> 3 days	3	453	0.66 [0.47, 0.81]	< 0.001	2.33	3	0	0.51	
Daily dose									0.14
Low (≤ 0.25 g/kg)	10	685	0.52 [0.42, 0.65]	< 0.001	5.12	9	0	0.82	
High (> 0.25 g/kg)	5	606	0.68 [0.55, 0.85]	< 0.001	7.06	4	29	0.22	
Total dose									0.03
Low (< 0.9 g/kg)	11	740	0.50 [0.40, 0.62]	< 0.001	7.39	10	0	0.69	
High (≥ 0.9 g/kg)	5	551	0.70 [0.56, 0.87]	0.002	3.80	4	0	0.43	
Type of control intervention									0.14
Placebo	14	1141	0.57 [0.48, 0.67]	< 0.001	11.26	13	0	0.59	
Human albumin solution	5	389	0.74 [0.54, 1.01]	0.05	1.56	4	0	0.45	
Follow-up duration									0.21
≤ 28 days	11	1135	0.64 [0.54, 0.76]	< 0.001	10.14	10	1	0.43	
> 28 days	3	158	0.35 [0.17, 0.71]	0.004	3.42	2	42	0.18	
ICU days	5	237	0.56 [0.40, 0.77]	< 0.001	1.87	4	0	0.76	
Study design									0.27
Randomized controlled trial	15	978	0.65 [0.53, 0.78]	< 0.001	10.72	14	0	0.71	
Cohort study	4	552	0.35 [0.25, 0.50]	< 0.001	4.80	3	37	0.19	
Publication year									0.21
Old studies (before 2005)	10	485	0.52 [0.40, 0.68]	< 0.001	6.38	9	0	0.70	
Recent studies (from 2005)	9	1045	0.64 [0.54, 0.76]	< 0.001	10.05	8	20	0.26	

*K* number of studies, *N* number of participants, *ICU* intensive care unit, *RR* relative risk, *CI* confidence interval

### Secondary outcomes

**Fig. 5:**
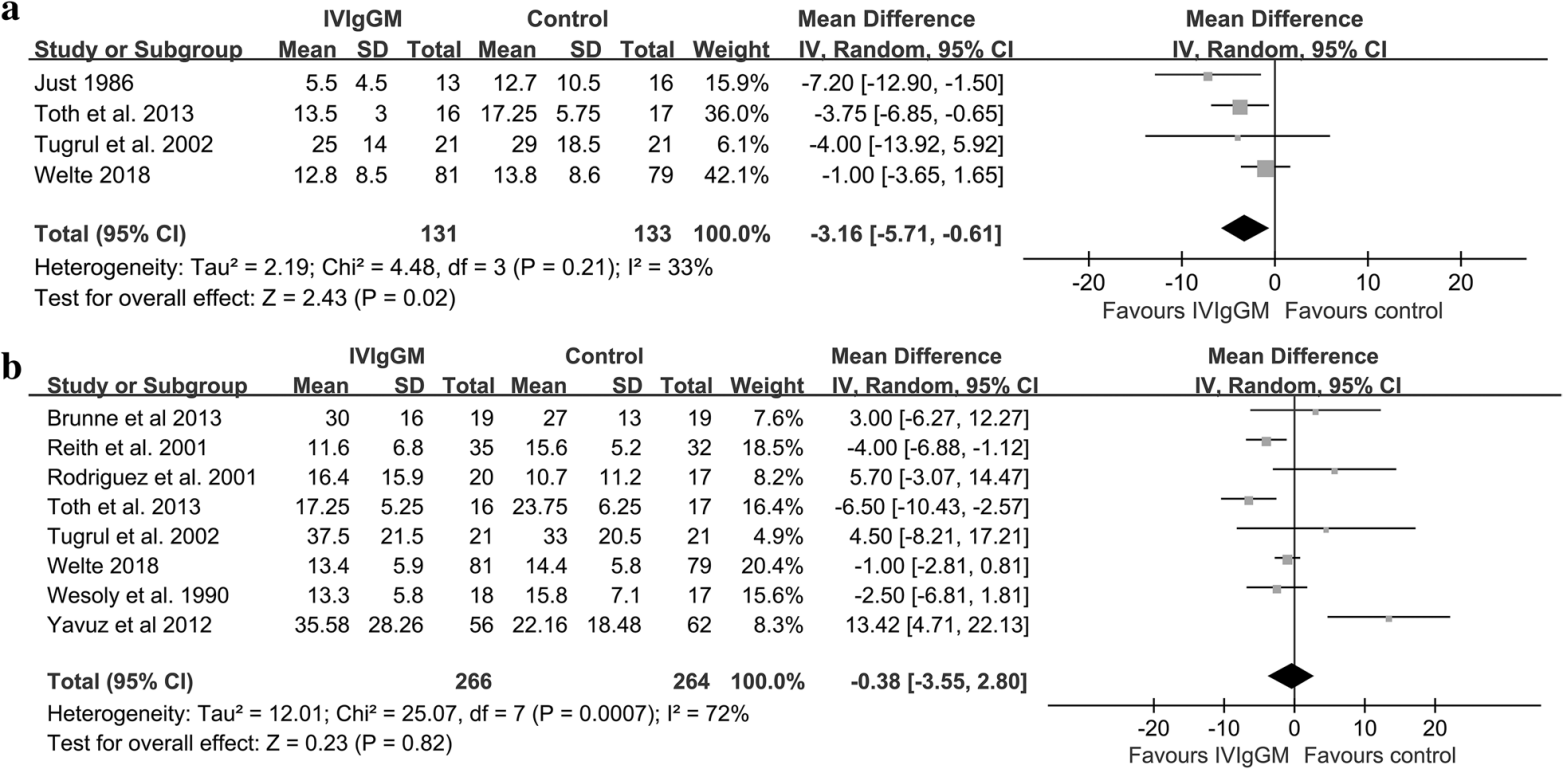
Forest plot for length of mechanical ventilation **a** and ICU length of stay **b** after IVIgGM administration

**Fig. 6:**
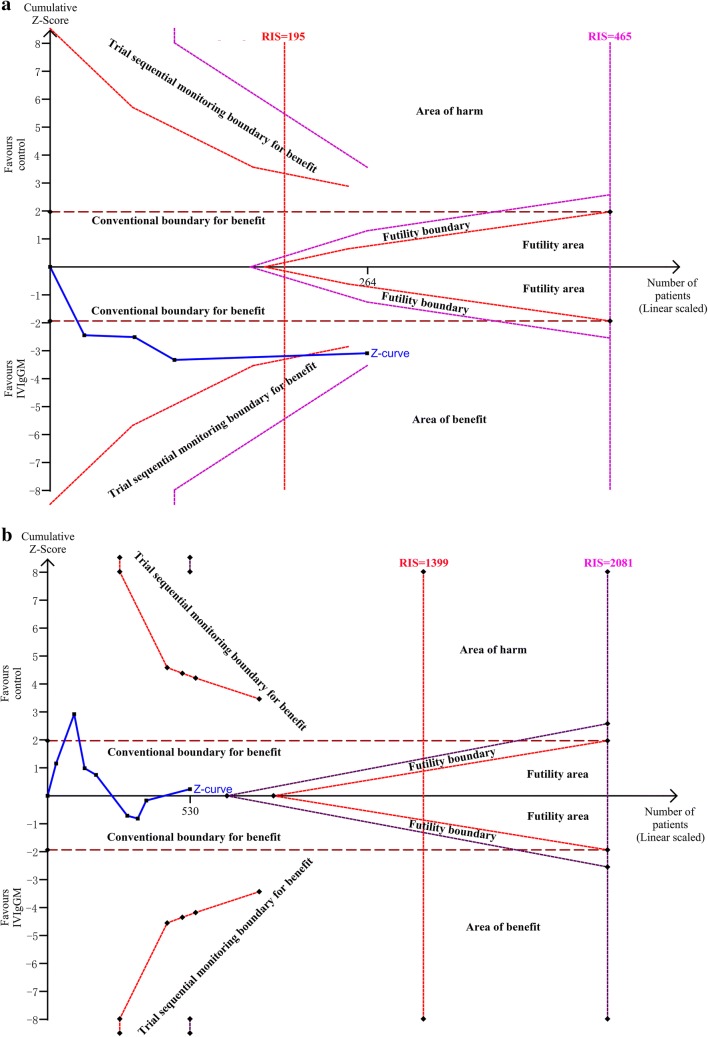
**a** Trial sequential analysis for length of mechanical ventilation in trials: A diversity-adjusted information size of 80 circuits was calculated on the basis of a MD of − 3.16, variance of 53.52, *I*^2^ = 33%, *α* = 5% (two-sided) and *β* = 20%. The cumulative *Z*-curve crosses the trial sequential monitoring boundary for benefit and reaches the required information size. **b** Trial sequential analysis for ICU LOS in trials: A diversity-adjusted information size of 1191 circuits was calculated on the basis of a MD of − 0.38, variance of 10.36, *I*^2^ = 72%, *α* = 5% (two-sided) and *β* = 20%. The cumulative *Z*-curve did not cross the conventional boundary for benefits and did not enter the futility boundary

The length of mechanical ventilation was significantly shorter in IVIgGM group than in the control group, with a mean difference of − 3.16 days (95% CI − 5.71 to − 0.61 days; *I*^2^ = 33%) (Fig. 5a). TSA showed that the cumulative Z-curves crossed both the conventional boundary and the trial sequential monitoring boundary. Thus, further trials were unlikely to change the conclusion (Fig. 6a). However, pooled analysis demonstrated no significant differences in the ICU LOS between the two groups, with a mean standard difference of 0.38 days (95% CI − 3.55 to 2.80 days; *I*^2^ = 72%) (Fig. 5b). TSA analysis indicated the cumulative *Z*-curve did not cross the conventional boundary for benefits and did not enter the futility boundary. A TSA sensitivity analysis that included all trials indicated that the diversity-adjusted required information size was 1399 (Fig. 6b).

### Publication bias

**Fig. 7:**
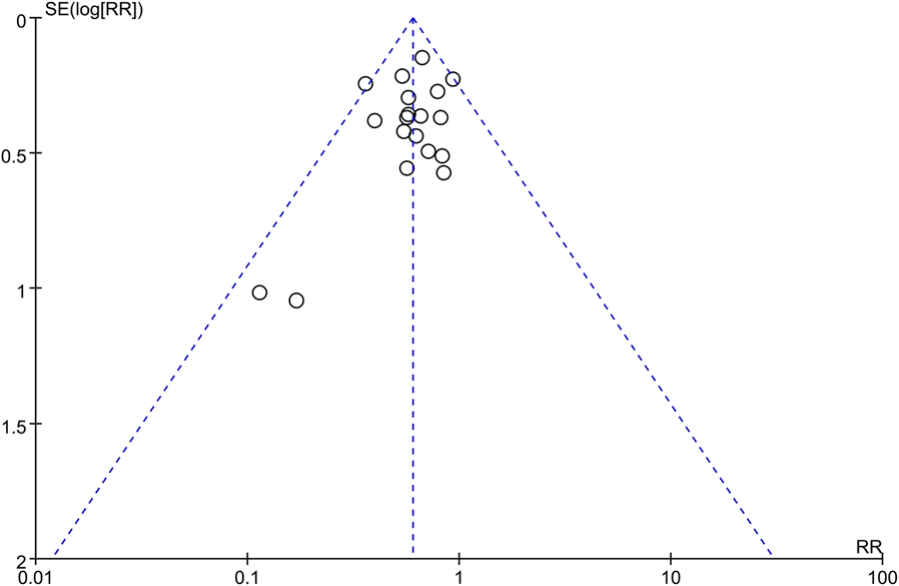
Assessment of publication bias using a funnel plot

Assessment of potential publication bias for the primary outcome (all-cause mortality) showed no bias among the included trials, as indicated by the presence of all results within the funnel (Fig. 7).

### Grade

The GRADE level of evidence for survival benefits from IVIgGM and length of mechanical ventilation was low and the level of evidence for ICU LOS was very low (Table 3).

**Table 3:** Summary of findings table

Patient or population: patients with Sepsis or septic shock					
Settings: Intensive care medicine					
Intervention: IVIgGM					
Comparison: Control					

Outcomes	Illustrative comparative risks* (95% CI)		Relative effect(95% CI)	No of Participants(studies)	Quality of the evidence(GRADE)
	Assumed risk	Corresponding risk			
	*Control*	*IVIgGM*			
New OutcomeFollow-up: 12-70 days	*Study population*		*RR 0.60* (0.52 to 0.69)	1530 (19 studies)	⊕⊕⊝⊝ *low* ^1^
	*429 per 1000*	*258 per 1000* (223 to 296)			
	*Moderate*				
	*412 per 1000*	*247 per 1000* (214 to 284)			
Length of mechanical ventilation	The mean length of mechanical ventilation in the intervention groups was *3.16 lower (5.71 lower to 0.61 lower)*			264 (4 studies)	⊕⊕⊝⊝ *low* ^1^
Length of stay on ICU	The mean length of stay on ICU in the intervention groups was *0.38 higher* (3.55 lower to 2.80 higher)			530 (8 studies)	⊕⊝⊝⊝ *very low* ^1^

*The basis for the assumed risk (e.g. the median control group risk across studies) is provided in footnotes. The corresponding risk (and its 95% confidence interval) is based on the assumed risk in the comparison group and the relative effect of the intervention (and its 95% CI)

*CI* confidence interval, *RR* risk ratio, *ICU* intensive care medicine

GRADE Working Group grades of evidence

High quality: Further research is very unlikely to change our confidence in the estimate of effect

Moderate quality: Further research is likely to have an important impact on our confidence in the estimate of effect and may change the estimate

Low quality: Further research is very likely to have an important impact on our confidence in the estimate of effect and is likely to change the estimate

Very low quality: We are very uncertain about the estimate

## Discussion

The present meta-analysis, which included 15 RCTs, involving 712 patients, and four cohort studies, involving 818 patients, assessed the use of IVIgGM preparations in adults with sepsis. IVIgGM administration significantly reduced mortality rates, with an RR of 0.60 (95% CI 0.52–0.69). Subgroup analysis showed that these results were generally consistent, regardless of duration of treatment, daily dose, total dose, variety of disease severity scores, follow-up duration, study design and year of publication. However, use of IVIgGM shortens mechanical ventilation days but not ICU LOS.

This systematic review and meta-analysis have a number of methodological strengths. The research question was focused to include a specific clinically relevant population and a specific intervention. First, the protocol of this study was registered on PROSPERO. A registered protocol may increase the transparency and quality of meta-analyses. Second, the present study took account of disease severity grades for subgroup analysis and included length of mechanical ventilation and ICU LOS as an outcome. Third, TSA was used to assess the risk of random errors (spurious findings), with results supporting the contention that a 20% relative increase or decrease in all-cause mortality can be confidently excluded. Finally, we provided the evidence body level using the GRADE approach, which classifies the conclusions of studies as having high, moderate, low, or very low quality of evidence.

Several recent systematic reviews and/or meta-analyses have evaluated the effects of IVIG on outcomes in patients with sepsis [10, 32–37], but these analyses have yielded conflicting results. Several previous meta-analyses found that treatment with IVIgGM more consistently reduced mortality than treatment with standard polyclonal IgG [10]. Because of the limited number of articles, meta-analyses focused on a single type of IVIG (IVIgGM) had been not conducted. A recent review [37] summarizing current data on the established clinical uses of IVIgGM in patients with sepsis did not statistically pool available data to analyze whether IVIgGM treatment was associated with patient mortality.

The results of this systematic review and meta-analysis indicate that, although IVIgGM did not shorten ICU LOS, it significantly reduced patient mortality rates and shorten ventilation days. In addition, our TSA of the primary outcome provided conclusive evidence that further trials were not required. Because many of these studies included mixed patient cohorts and patients at high risk, GRADE deemed the certainty of the body of evidence as low for both benefits and IVIgGM. Based on these findings, our center will be cautious in treating sepsis patients with IVIgGM as adjuvant therapy.

The choice of the control intervention had a potential impact on the overall treatment effect. In contrast to one meta-analysis [36] and in accordance with another [34], a subgroup analysis based on type of control arm found that IVIgGM did not significantly reduce mortality rates when compared with HAS, but did significantly reduce mortality rates when compared with no treatment. Moreover, the effect of IVIgGM tended to be smaller and less consistent when compared with HAS than when compared with no treatment, suggesting that HAS may have had a biological effect. HAS has oncotic, carrier, antioxidant and anti-inflammatory properties and has been associated with a significant reduction in mortality rates in patients with septic shock [38]. Alternatively, the use of HAS may be an indicator of more appropriate blinding, thus being associated with an overall higher study quality and lower risk of bias.

A meta-analysis [34] has shown that different dosing regimens and durations of treatment appeared to affect the mortality rates. Our meta-regression analysis revealed that lower daily and total doses of IVIgGM tended to be associated with greater and more consistent reductions in mortality rate, although these differences were not statistically significant. As previously described [35], it is difficult to identify a clinical rationale for these associations. Thus, these results should be interpreted with caution, especially because of the high variability in study characteristics and patients differing in the severity of sepsis.

Timing of IVIg administration is controversial. A recent study investigated the protective association between endogenous IVIg level and sepsis mortality [39]. They found there is a risky level of immunoglobulins attribute to mortality in moderate (SOFA < 8) but not severe sepsis(SOFA ≥ 8), which drove researchers concerning much about IVIg substitution is worthy in different populations. Our subgroup analysis reveals that IVIgGM is beneficial for sepsis regardless of the disease severity (higher SOFA score, shock, or not). Although we deem believe more evidence should be added in, the present results conveyed a certain of confidence for clinicians to treat sepsis patients.

For secondary outcomes, as the heterogeneity of available data, this meta-analysis did not find a significant reduction of ICU LOS after IVIgGM administration. ICU stay is a heterogeneous variable per se. Numerous social factors complicated with medical resources control influent the length of ICU stay [40]. More reliable parameters like ventilation duration or free of ventilation days (VFDs) should be considered in clinical trials. With the available data extracted from literatures, ventilations days are significantly reduced in IVIgGM group patients, however, IVIgGM use did not increase VFDs (Additional file 6: Figure S3). Further research should evaluate this respiratory benefit.

This systematic review had several limitations. First, study characteristics varied widely, including in duration and dose of IVIgGM and control interventions. However, our meta-analysis verified that IVIgGM treatment of adult patients with sepsis has a consistent rationale and was associated with a reduction in the relative risk of mortality, with low heterogeneity (*I*^2^ = 0). Second, the number of observational studies was relatively small, and there were few latest RCTs, indicating a need for large, multi-centered RCTs to support the present results. This study included data from the last 30 years. There have been many improvements in intensive care over the last half-century, including wider ranges of patients, as determined by both age and comorbidities, now treated within a critical care environment, suggesting a possibility of bias. Although the inclusion criteria for studies may not have changed, the populations in these studies have altered. Many patients not considered appropriate for critical care in the 1980s and 1990s now constitute the majority of patients in many critical care units in developed countries. Our exploration of subgroup hypotheses, including the era in which the studies were conducted, and the failure to identify any effect modification. Furthermore, the latest CIGMA study (Welte et al. 2018) [14] is strongly consistent with the final results, which markedly diminishes this concern. Fourth, all included studies enrolled patients based on previous diagnostic criteria for sepsis, as patients diagnosed by Sepsis-3 is more severe than Sepsis-2. Although the subgroup analysis is consistent with overall effect (sepsis vs severe sepsis or septic shock), we suspect that using the new Sepsis-3 criteria could change the efficacy of IVIgGM. Fifth, the reduction in mortality rate associated with IVIgGM was greater in studies performed among populations with higher baseline risk. Many patient-related factors may influence the clinical effect of IVIgGM therapy, such as an underlying state of immunosuppression, basal levels of endogenous Ig, time from sepsis diagnosis and IVIgGM administration or concurrent treatments [41–43]. Finally, it was not possible to analyze IVIgGM-related adverse effects, as these were reported by few of the included studies included. The use of IVIgGM, especially sucrose-stabilized IVIG preparations, has been associated with the onset of acute renal failure due to osmotic nephrosis in the proximal tubules [44, 45]. A specific review is required to clarify this point.

## Conclusion

The administration of IVIgGM to adult septic patients may have a rationale and may be associated with reduced mortality rates. Because current evidence is insufficient and too low quality to support the widespread use of IVIgGM as adjunctive therapy for sepsis, large-scale high-quality RCTs, using recently published Sepsis 3 definitions, are warranted to assess the benefits and potential uses of IVIgGM in the treatment of sepsis syndrome.

## Additional files

**Additional file 1: Table S1.** Primary and secondary outcomes of the studies included in the meta-analysis.

**Additional file 2: Table S2.** Quality Assessment for Randomized Controlled Trials.

**Additional file 3: Table S3.** Quality Assessment With Newcastle–Ottawa Scale for cohort study.

**Additional file 4: Figure S1.** Subgroup analysis-sepsis vs severe sepsis or septic shock, evaluating survival benefit of intravenous IgM-enriched immunoglobulin (IVIgGM).

**Additional file 5: Figure S2.** Subgroup analysis-severity scores, evaluating survival benefit of intravenous IgM-enriched immunoglobulin (IVIgGM).

**Additional file 6: Figure S3.** Forest plot for ventilation free days (VFDs) after intravenous IgM-enriched immunoglobulin (IVIgGM).

## Abbreviations

IVIgGM: intravenous IgM-enriched immunoglobulin
TSA: trial sequential analysis
GRADE: Grading of Recommendations Assessment, Development, and Evaluation
CI: confidence interval
SSC: surviving sepsis campaign
IVIG: intravenous immunoglobulins
APACHE: Acute Physiology and Chronic Health Evaluation
SOFA: Sequential Organ Failure Assessment
LOS: length of stay
ICU: intensive care unit
RCTs: randomized controlled trials
RR: risk ratio
WMD: weighted mean difference
MRA: meta-regression analysis
VFDs: ventilation-free days
N: number
NR: not reported
HAS: human albumin solution
ROS: retrospective observational study
POS: prospective observational study
REC: Representative of Exposed Cohort
SNC: Selection of Nonexposed Cohort
AE: Ascertainment of Exposed
AOI: Absence of Outcome of Interest
