# Supplementary material for: The clinical efficacy of intravenous IgM-enriched immunoglobulin (pentaglobin) in sepsis or septic shock: a meta-analysis with trial sequential analysis
Source: Ann Intensive Care. 2019 Feb 6;9:27. doi: 10.1186/s13613-019-0501-3 (PMC6365591; doi:10.1186/s13613-019-0501-3)
Supplement: Supplementary file 1 — Additional file 1: Table S1. Primary and secondary outcomes of the studies included in the meta-analysis. [file 13613_2019_501_MOESM1_ESM.docx]

**Table S1.** Primary and secondary outcomes of the studies included in the meta-analysis.

| Study | Mortality rate (%) | | length of mechanical ventilation | | Length of stay on ICU | |
| --- | --- | --- | --- | --- | --- | --- |
|  | **IVIgGM group** | **Control group** | **IVIgGM group** | **Control group** | **IVIgGM group** | **Control group** |
| Behre et al. 1995 | 9/30 | 10/22 | NR | NR | NR | NR |
| Brunne et al 2013 | 5/19 | 6/19 | NR | NR | 30 ± 16 | 27 ± 13 |
| Buda et al 2005 | 5/22 | 16/44 | NR | NR | NR | NR |
| Cavazzuti et al 2014 | 23/92 | 35/76 | NR | NR | NR | NR |
| Giamarellos-Bourboulis  et al 2016 | 39/100 | 58/100 | NR | NR | NR | NR |
| Hentrich et al. 2006 | 27/103 | 29/103 | NR | NR | NR | NR |
| Just et al 1986 | 6/13 | 9/16 | 5.5±2.5 | 12.7±6.5 | NR | NR |
| Karatzas et al. 2002 | 8/34 | 14/34 | NR | NR |  |  |
| Reith et al. 2001 | 7/35 | 16/32 | NR | NR | 11.6±6.8 | 15.6±5.2 |
| Rodriguez et al. 2001 | 1/20 | 5/17 | NR | NR | 16.4±15.9 | 10.7±11.2 |
| Rodriguez et al. 2005 | 8/29 | 13/27 | NR | NR |  |  |
| Schedel et al. 1991 | 1/27 | 9/28 | NR | NR |  |  |
| Spannbrucker et al. 1987 | 4/25 | 7/25 | NR | NR |  |  |
| Toth et al. 2013 | 4/16 | 5/17 | 13.5±3 | 17.25±5.75 | 17.25±5.25 | 23.75±6.25 |
| Tugrul et al. 2002 | 5/21 | 7/21 | 25±14 | 29±18.5 | 37.5±21.5 | 33±20.5 |
| Vogel et al 1988 | 6/25 | 11/25 | NR | NR |  |  |
| Welte et al 2018 | 18/18 | 22/79 | 12.8±8.5 | 13.8±8.6 | 13.4±5.9 | 14.4±5.8 |
| Wesoly et al. 1990 | 8/18 | 13/17 | NR | NR | 13.3±5.8 | 15.8±7.1 |
| Yavuz et al 2012 | 14/56 | 43/62 | NR | NR | 35.58±28.26 | 22.16±18.48 |

Data are reported as mean ± standard deviation or median. Abbreviations: NR= not reported,
