# Supplementary material for: The clinical efficacy of intravenous IgM-enriched immunoglobulin (pentaglobin) in sepsis or septic shock: a meta-analysis with trial sequential analysis
Source: Ann Intensive Care. 2019 Feb 6;9:27. doi: 10.1186/s13613-019-0501-3 (PMC6365591; doi:10.1186/s13613-019-0501-3)
Supplement: Supplementary file 2 — Additional file 2: Table S2. Quality Assessment for Randomized Controlled Trials. [file 13613_2019_501_MOESM2_ESM.docx]

**Table S2.** Quality Assessment for Randomized Controlled Trials

| Study | Sequence Generation | Allocation Concealment | Blinding | Incomplete outcome data addressed | Free of selective reporting | Free of other Bias | Concurrent therapies similar | Overall risk of bias |
| --- | --- | --- | --- | --- | --- | --- | --- | --- |
| Behre et al. 1995 | Unclear | Unclear | Unclear | Unclear | Yes | Yes | Yes | High |
| Brunne et al 2013 | Yes | Yes | Yes | Unclear | Yes | Yes | Yes | Low |
| Hentrich et al. 2006 | Yes | Yes | Yes | Unclear | Yes | Yes | Yes | Low |
| Just et al 1986 | Unclear | Unclear | Unclear | Unclear | Yes | Yes | Yes | High |
| Karatzas et al. 2002 | Unclear | Unclear | Unclear | Unclear | Yes | Yes | Yes | High |
| Reith et al. 2001 | Yes | Unclear | Unclear | Unclear | Yes | Yes | Yes | Moderate |
| Rodriguez et al. 2001 | Yes | Yes | Yes | Yes | Yes | Yes | Yes | Low |
| Rodriguez et al. 2005 | Yes | Yes | Yes | Unclear | Yes | Yes | Yes | Low |
| Schedel et al. 1991 | Yes | Yes | Yes | Unclear | Yes | Yes | Yes | Low |
| Spannbrucker et al. 1987 | Yes | Unclear | Unclear | Unclear | Yes | Yes | Yes | Moderate |
| Toth et al^.^ 2013 | Yes | Yes | Unclear | Unclear | Yes | Yes | Yes | Moderate |
| Tugrul et al. 2002 | Yes | Unclear | Unclear | Unclear | Yes | Yes | Yes | Moderate |
| Vogel et al 1988 | Unclear | Unclear | Unclear | Unclear | Yes | Yes | Yes | High |
| Welte et al 2018 | Yes | Yes | Yes | Yes | Yes | Yes | Yes | Low |
| Wesoly et al. 1990 | Yes | Unclear | Unclear | Unclear | Yes | Yes | Yes | Moderate |
