# Supplementary material for: The clinical efficacy of intravenous IgM-enriched immunoglobulin (pentaglobin) in sepsis or septic shock: a meta-analysis with trial sequential analysis
Source: Ann Intensive Care. 2019 Feb 6;9:27. doi: 10.1186/s13613-019-0501-3 (PMC6365591; doi:10.1186/s13613-019-0501-3)
Supplement: Supplementary file 3 — Additional file 3: Table S3. Quality Assessment With Newcastle–Ottawa Scale for cohort study. [file 13613_2019_501_MOESM3_ESM.docx]

**Table S3. *Quality Assessment With Newcastle Ottawa Scale for cohort study***

| Study | **Selection** | | | | **Comparability** | **Outcome** | | |  |
| --- | --- | --- | --- | --- | --- | --- | --- | --- | --- |
|  | **REC** | **SNC** | **AE** | **AOI** | **Design and**  **Analysis** | **Assessment** | **Enough**  **Follow-up** | **Adequate**  **Follow-up** | **Score** |
| Buda et al 2005 | **☆** | **☆** | **☆** | **☆** | **☆☆** | **☆** | **☆** | **☆** | **9** |
| Cavazzuti et al 2014 | **☆** | **☆** | **☆** | **☆** | **☆☆** | **☆** | **☆** | **☆** | **9** |
| Giamarellos-  Bourboulis  et al 2016 | **☆** | **☆** | **☆** | **☆** | **☆☆** | **☆** | **☆** | **☆** | **9** |
| Yavuz et al 2012 | **☆** | **☆** | **☆** | **☆** | **☆** | **☆** | **☆** | **☆** | **8** |

**REC=Representative of Exposed Cohort , SEC=Selection of Nonexposed Cohort，AE=Ascertainment of Exposed, AOI=Absence of Outcome of Interest, star (✩) was allocated to a particular item when it was adequately reported and addressed. The item “comparability” could be allocated with a maximum of two stars. Dashes indicate this item was not adequately reported or addressed.**
