# Supplementary figures and images for: The clinical efficacy of intravenous IgM-enriched immunoglobulin (pentaglobin) in sepsis or septic shock: a meta-analysis with trial sequential analysis
Source: Ann Intensive Care. 2019 Feb 6;9:27. doi: 10.1186/s13613-019-0501-3 (PMC6365591; doi:10.1186/s13613-019-0501-3)

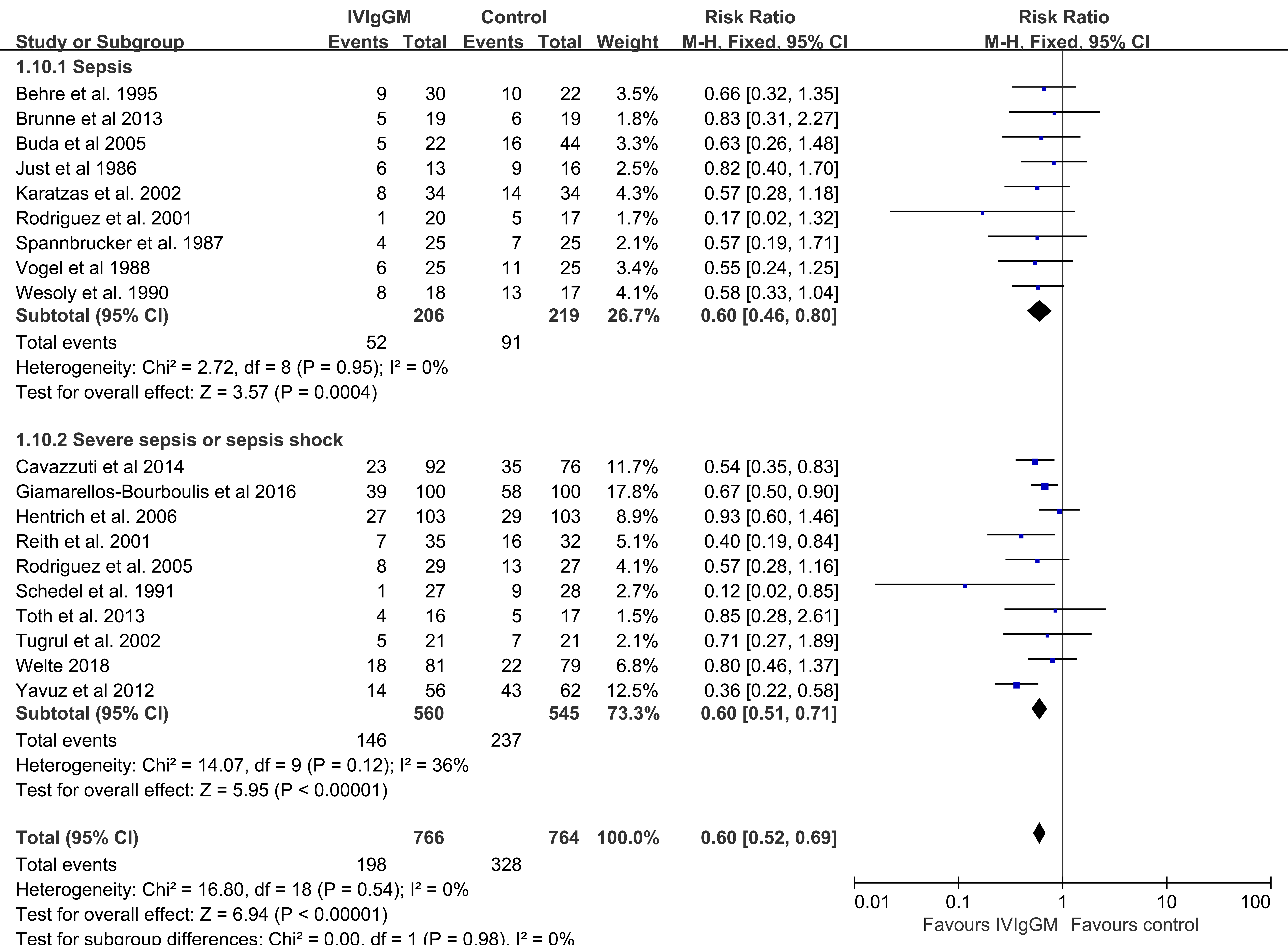

Supplement: Supplementary file 4 — Additional file 4: Figure S1. Subgroup analysis-sepsis vs severe sepsis or septic shock, evaluating survival benefit of intravenous IgM-enriched immunoglobulin (IVIgGM). [file 13613_2019_501_MOESM4_ESM.tif]

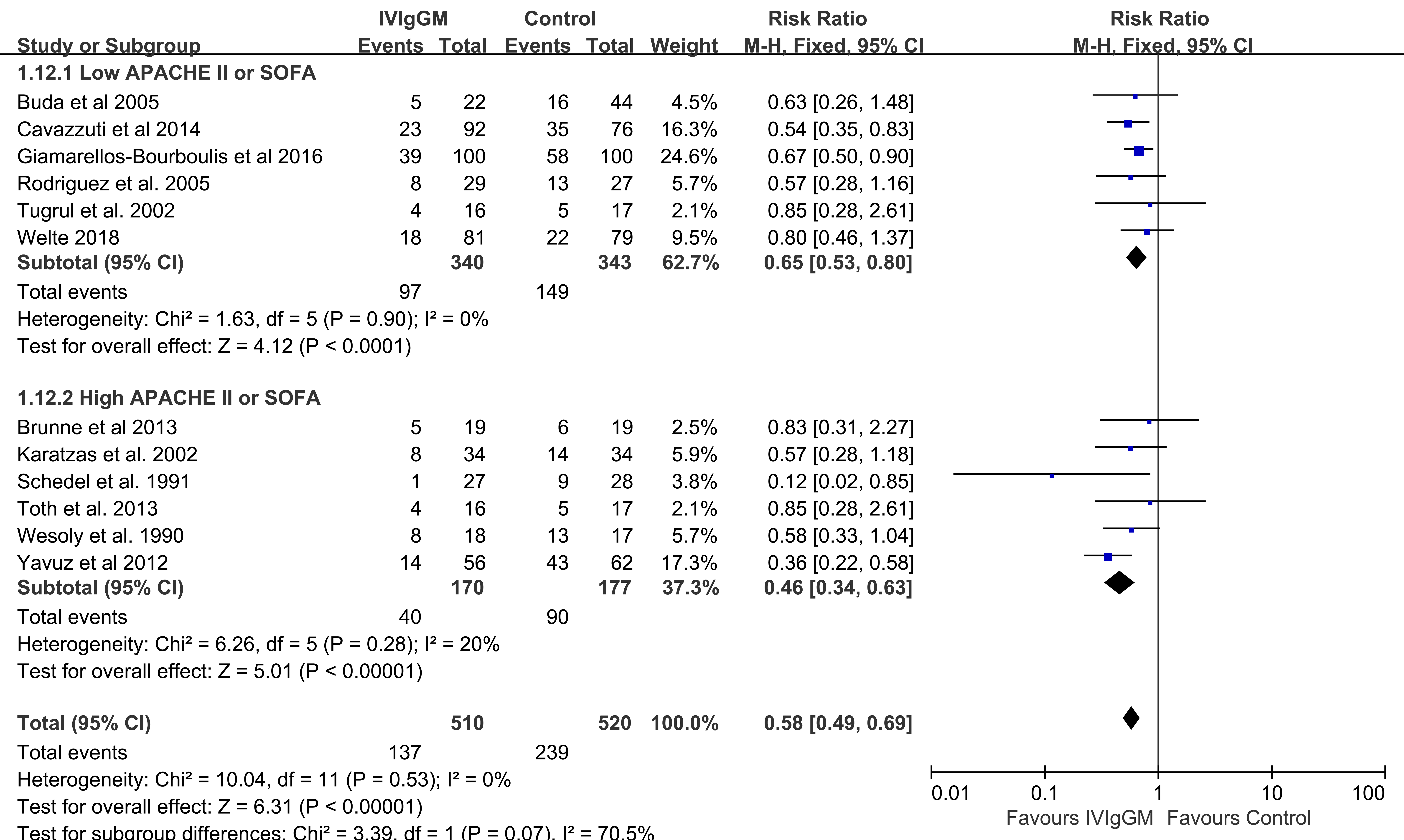

Supplement: Supplementary file 5 — Additional file 5: Figure S2. Subgroup analysis-severity scores, evaluating survival benefit of intravenous IgM-enriched immunoglobulin (IVIgGM). [file 13613_2019_501_MOESM5_ESM.tif]

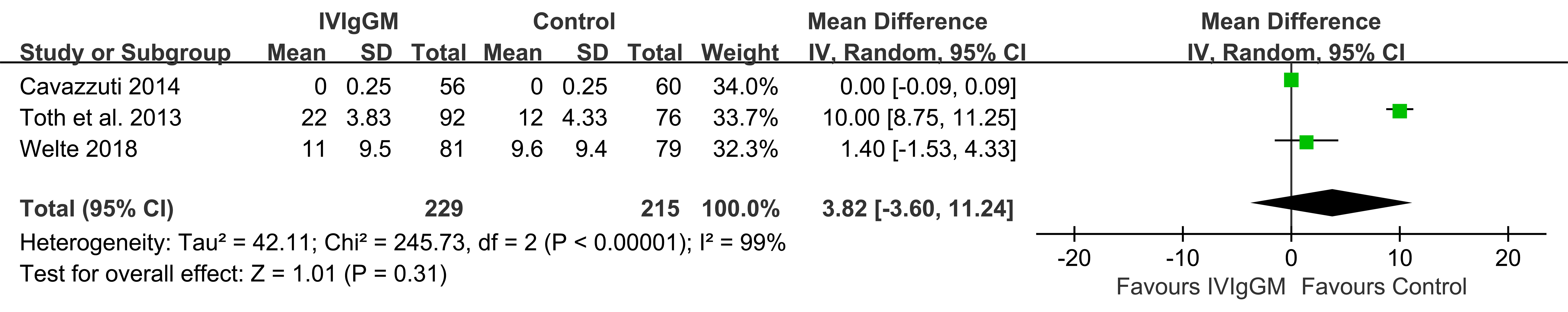

Supplement: Supplementary file 6 — Additional file 6: Figure S3. Forest plot for ventilation free days (VFDs) after intravenous IgM-enriched immunoglobulin (IVIgGM). [file 13613_2019_501_MOESM6_ESM.tif]
